# A Surface-Enhanced Raman Scattering Sensor Integrated with Battery-Controlled Fluidic Device for Capture and Detection of Trace Small Molecules

**DOI:** 10.1038/srep12865

**Published:** 2015-08-04

**Authors:** Qitao Zhou, Guowen Meng, Peng Zheng, Scott Cushing, Nianqiang Wu, Qing Huang, Chuhong Zhu, Zhuo Zhang, Zhiwei Wang

**Affiliations:** 1 Key Laboratory of Materials Physics, and Anhui Key Laboratory of Nanomaterials and Nanostructures, Institute of Solid State Physics, Chinese Academy of Sciences, Hefei 230031, P. R. China; 2 University of Science & Technology of China, Hefei 230026, P. R. China; 3 Department of Mechanical & Aerospace Engineering, West Virginia University, P.O. Box 6106, Morgantown, WV 26506, USA; 4 Department of Physics and Astronomy, West Virginia University, Morgantown, WV 26506, USA; 5 Key Laboratory of Ion Beam Bioengineering, Chinese Academy of Sciences, Hefei 230031, P. R. China

## Abstract

For surface-enhanced Raman scattering (SERS) sensors, one of the important issues is the development of substrates not only with high SERS-activity but also with strong ability to capture analytes. However, it is difficult to achieve the two goals simultaneously especially when detecting small molecules. Herein a compact battery-controlled nanostructure-assembled SERS system has been demonstrated for capture and detection of trace small molecule pollutants in water. In this SERS fluidic system, an electrical heating constantan wire covered with the vertically aligned ZnO nanotapers decorated with Ag-nanoparticles is inserted into a glass capillary. A mixture of thermo-responsive microgels, Au-nanorods colloids and analyte solution is then filled into the remnant space of the capillary. When the system is heated by switching on the battery, the thermo-responsive microgels shrink, which immobilizes the analyte and drives the Au-nanorod close to each other and close to the Ag-ZnO nanotapers. This process has also created high-density “hot spots” due to multi-type plasmonic couplings in three-dimensional space, amplifying the SERS signal. This integrated device has been successfully used to measure methyl parathion in lake water, showing a great potential in detection of aquatic pollutants.

Surface-enhanced Raman scattering (SERS) has a wide range of applications in surface^1^,^2^, biological^3^,^4^ and analytical^5^ sciences especially for rapid analysis of trace (bio-) chemicals^6^ due to its high sensitivity, fast response and fingerprint characteristics of SERS signals. It is generally accepted that noble metal nanostructures are able to amplify the SERS signals by localized surface plasmon resonance (LSPR)-induced electromagnetic field enhancement, while semiconducting nanostructures exhibit chemical enhancement effect ascribed to the charge transfer between the semiconductor and the analyte molecules^7^,^8^,^9^,^10^.

When a compact SERS device is used for real-time detection of trace toxic pollutants in the environment, the key to success of SERS analysis is to construct a substrate that has not only high SERS activity but also strong capability of capturing the analyte^11^,^12^. However, it is difficult to achieve the two goals simultaneously especially when detecting small molecules. When detecting macromolecules such as proteins, the antibody immobilized on the SERS substrate is employed to capture the antigen (analyte)^13^. Unfortunately, limited antibody and other molecular recognition probes are available for capturing small molecules. To enable the detection of small molecules in the environmental samples, sealed apparatuses have been reported to prevent from volatilization, in which the plasmonic Ag/Au nanoparticles (NPs) and the analytes were blended and confined/enclosed in a microfluidic system^14^,^15^,^16^; and the analyte solution was injected into the microfluidic channel decorated with SERS active Ag-covered nanopillar arrays^17^. However, the plasmonic Ag/Au NPs or the Ag-covered nanopillars can only capture the nearby analytes for SERS measurement via chemi/physi-sorption, which limits the sensitivity of the SERS device. In order to capture more analyte effectively, thermo-responsive microgels were introduced into the SERS system. For example, poly (N-isopropylacrylamide) (pNIPAM) microgels were mixed with the plasmonic Au-NPs to form the Au-NPs@pNIPAM core@shell colloids. When the system was heated to a given temperature, the thermo-responsive pNIPAM shells were collapsed, leading to mechanically trapping of the analytes in the proximity to the plasmonic Au-NPs core for the enhanced SERS signal^18^. Recently the plasmonic nanogaps achieved by electrostatic forces have attracted many attentions^19^. In addition, the pNIPAM was also used as a thermo-responsive part in a nanogap tunable SERS substrate to trap the analyte to enhance the SERS signal^20^,^21^. However, it remains a challenge to develop an integrated SERS device for detection of small molecules in the environment. The present work for the first time presents an integrated compact device that is able to online capture and to detect small molecules in an aqueous solution.

## Results Section

**Figure 1:**
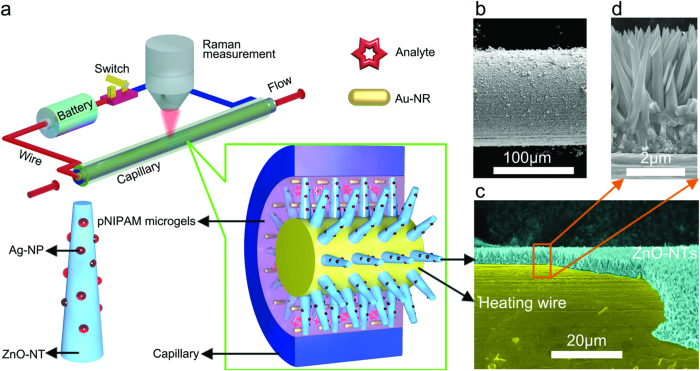
Schematic illustration of the battery-controlled composite SERS-based fluidic system and characterizations. (**a**) a battery-controlled SERS fluidic system. (**b**) SEM image of the constantan wire after electrodeposition of the ZnO-NTs. (**c**) and (**d**) SEM images of the ZnO-NTs covered constantan wire after removing part of ZnO-NTs and the close-up view. This figure was drawn by one of the coauthors (Qitao Zhou).

Figure 1a shows the compact battery-controlled hierarchical hetero-nanostructure-assembled fluidic SERS system. The device was built by the following procedure (Fig. S1). First, a radial ZnO-nanotaper (ZnO-NT) array was electrodeposited on an electric heating constantan-wire (denoted as ZnO-NTs@constantan-wire), then the high-density Ag-NPs were decorated onto each ZnO-NT (denoted as Ag-NPs@ZnO-NTs@constantan-wire). Next, the Ag-NPs@ZnO-NTs@constantan-wire was inserted into a glass capillary. Finally, the analyte solution, Au nanorods (Au-NRs) and the thermo-responsive pNIPAM microgels were mixed and injected into the capillary to fill the empty space. This integrated system has demonstrated much stronger SERS enhancement than the free-standing Au-NRs in the solution, or the Ag-NPs decorated on the ZnO-NT, or the Au-NRs coupled to the Ag-NPs grown on the ZnO-NT. This strongly suggests that the thermo-sensitive microgel plays a significant role in amplifying the SERS signal. In fact, when the batter-controlled circuit is switched on to heat the system to above 32 °C, the thermo-responsive pNIPAM microgels will shrink, leading to not only mechanical trapping of the analyte molecules much closer to the plasmonic Au-NRs and the Ag-NPs decorated on the ZnO-NTs but also abridging the spacing of the neighboring Au-NRs in the microgels, and that of the Au-NRs and their nearby Ag-NPs decorated on the ZnO-NTs, leading to the formation of high-density “hot spots” in the three-dimensional (3D) space.

**Figure 2:**
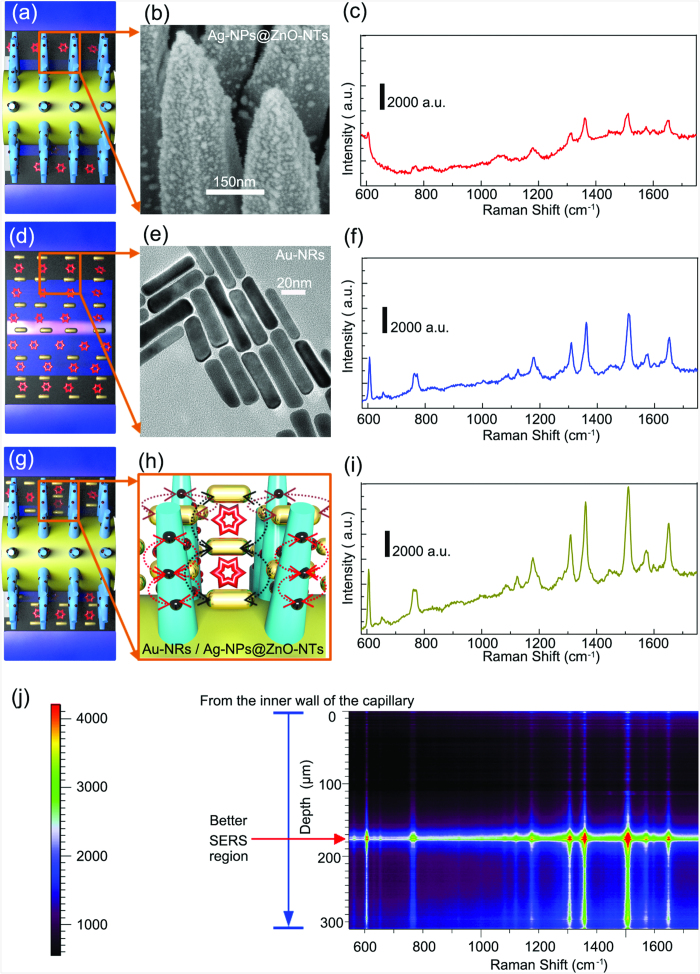
SERS spectra on different nano-building blocks. (**a**,**b**) Schematic of the Ag-NPs@ZnO-NTs@constantan-wire inside of a glass capillary surrounded by R6G solution and the corresponding SEM image of the Ag-NPs@ZnO-NTs covered on the constantan-wire, (**c**) SERS activity on the substrate shown in (**a**); (**d**,**e**) Schematic of a mixture of Au-NRs and R6G in a glass capillary and the corresponding TEM image of the Au-NRs, (**f**) SERS activity on the substrate shown in (**d**); (**g**,**h**) Schematic of the Ag-NPs@ZnO-NTs@constantan-wire inside of a glass capillary surrounded by mixture of Au-NRs and R6G, (**i**) SERS activity on the substrate shown in (**g**), SERS acquisition time: 30 s; (**j**) The depth SERS mapping was accompanied by adjusting the focus of incident light from inner wall of the capillary and 300 μm down. The depth SERS mapping was acquired with an integration time of 2 s. This figure was drawn by one of the coauthors (Qitao Zhou).

The naked glass capillary (with I.D. of 500 μm and O.D. of 700 μm) was about 10 cm long (Fig. S2a,b). The bare constantan-wire with a smooth surface was about 100 μm in a diameter (Fig. S2c,d). After electrodeposition of ZnO, cone-shaped ZnO-NTs in a height of 5 μm were radially grown on the constantan-wire surface (Fig. 1b–d). In order to identify the SERS contribution from different nano-building blocks in the composite SERS system, we examined the SERS-activity of the Ag-NPs@ZnO-NTs@constantan-wire in the glass capillary filled with Rhodamine 6G (R6G) solution (Fig. 2a–c), the mixture of Au-NRs and R6G solution in the glass capillary (Fig. 2d–f) and the Ag-NPs@ZnO-NTs@constantan-wire filled with the mixed Au-NRs and R6G solution in the glass capillary (Fig. 2g–i). The high-density radially distributed semiconducting ZnO-NTs were electrodeposited on the constantan wire, and a large quantity of plasmonic Ag-NPs with diameter about dozens of nanometers were decorated on each of the ZnO-NT (Fig. 2b and Fig. S3). As a result, the Ag-NPs@ZnO-NTs@constantan-wire system enabled the detection of 5 × 10^−7^ M R6G as shown in Fig. 2c. Similarly, the plasmonic Au-NRs (Fig. 2e) detected 5 × 10^−7^ M R6G (Fig. 2f). When the Au-NRs were filled in the space between the Ag-NPs@ZnO-NTs@constantan-wire and the inner wall of the glass capillary (Fig. 2g,h), the SERS intensity increased remarkably because it was a combination of the first two systems (shown in Fig. 2a,d). This system (Fig. 2g) had multiple-type plasmonic couplings among the neighboring Au-NRs in the solution, the neighboring Ag-NPs grown on ZnO-NT, the Ag-NPs grown on ZnO-NT and the nearby Au-NRs in solution. That is, high-density “hot spots” were created in the 3D space, leading to strong enhancement of SERS signal (Fig. 2i). It should be mentioned that the SERS signal intensity changed with the focused position of the incident light, as can be seen more clearly in the corresponding depth SERS mapping shown in Fig. 2j, achieved by adjusting the focus of the incident light from the inner wall of the capillary downward to 300 μm. There was an obvious region of better SERS activity about several microns, which almost matched the length of the ZnO-NTs (about 5 μm). This further indicated that the high SERS activity was ascribed to the multi-type plasmonic couplings. As a result, this compact microfluidic SERS system was able to measure R6G as low as 5 × 10^−9^ M (Fig. S4).

To control the multi-type plasmonic coupling induced SERS enhancement, the thermo-responsive pNIPAM microgels mixed with Au-NRs and the analyte were injected in the empty space between the Ag-NPs@ZnO-NTs@constantan-wire and the inner wall of the capillary. The heating experiments revealed that swelling and deswelling of thermo-responsive pNIPAM microgels had significant effect on the optical absorption of the Au-NRs embedded in pNIPAM microgels, which was revealed by the UV-Visible spectra recorded in a range from 20 °C (completely swollen) to 45 °C (completely collapsed) (Fig. S5). As the temperature drove the phase transformation of the pNIPAM, the hydrogels were collapsed as the temperature increased, creating a shift of the LSPR band from 821 nm to 849 nm as shown in Fig. S5.

**Figure 3:**
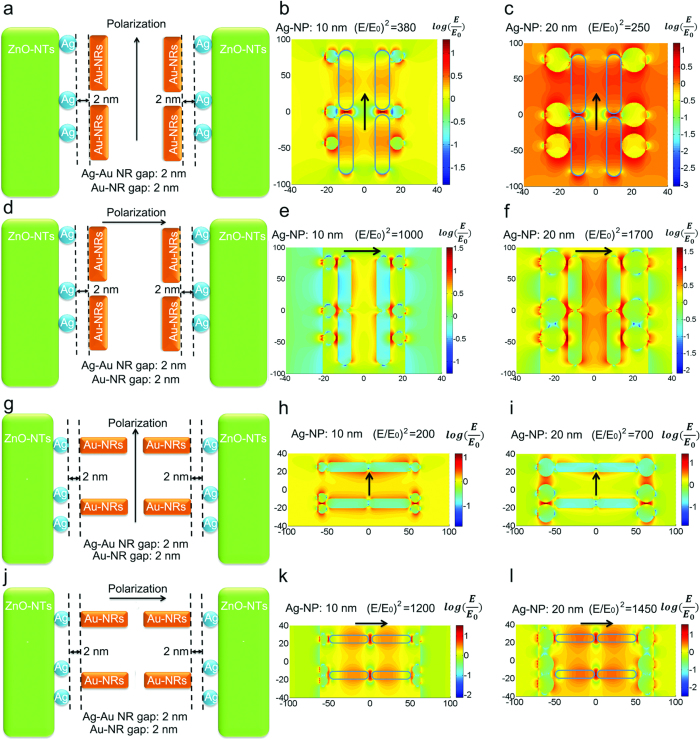
FDTD simulation results. FDTD simulations of the electric field distributions for ZnO-NTs decorated with Ag-NPs coupled with Au-NRs in a parallel alignment (**a**–**f**) and a perpendicular alignment (**g**–**l**). The incident polarization is in two different directions, (**a**,**j**) and (**d**,**g**) parallel and perpendicular to the nanorod. The |E/E_0_|^2^, the field enhancement normalized to the input field, for (**b**,**e**) and (**h**,**k**) a 2 nm gap with 10 nm Ag-NP and (**c**,**f**) and (**i**,**l**) a 2 nm gap with 20 nm Ag-NP is shown on a log-scale.

The red shift could come from two sources. First, the refractive index of the hydrogel increases with temperature, which redshifts the LSPR band^22^. Second, as the distance between the neighboring Au-NRs decreases, the LSPR modes will hybridize, leading to a redshift in the absorption^23^,^24^,^25^. The finite-difference time-domain (FDTD) simulation has confirmed that both effects lead to equal magnitude shifts, comparing Figs S6 and S7, presenting a delineation of each factor from the UV-Vis spectrum (Fig. S5). Although the refractive index and the proximity based shifts coexist, only changes in the separation distance will lead to a largely enhanced local electromagnetic (EM) field, amplifying the SERS signal. The ZnO-NTs decorated with Ag-NPs had a local electric field enhancement |E/E_0_|^2^ of 190 for a 10 nm Ag-NP and 100 for a 20 nm Ag-NP (Fig. S8). The Au-NRs had a peak enhancement of |E/E_0_|^2^ of 400 under the 633 nm excitation after being brought into contact end to end with a gap of 2 nm. The enhancement for several separation distances within the range estimated from the UV-Vis absorption is shown in Fig. S9. For side-side alignment, the coupled LSPR peak is off-resonance at 633 nm, and therefore does not have a large effect. The exact experimental configuration cannot be replicated for the Ag-NP, Au-NR, and ZnO-NT system after heating, so instead two cases are tested of which linear combinations can form all possible couplings. For Au-NR parallel to the Ag-NPs coated ZnO-NT, the field enhancement |E/E_0_|^2^ was as high as 1700 for the smallest separation distance and bigger Ag-NP size (Fig. 3f). For Au-NRs perpendicular to the ZnO-NT, the field enhancement |E/E_0_|^2^ was as high as 1450 (Fig. 3l). While the actual field enhancement will vary based on separation distance, alignment, and Ag-NP size, the FDTD simulations show that the increased Raman enhancement in the combined geometry originates in the close proximity and hybridization of the Ag-NP and Au-NR LSPR.

**Figure 4:**
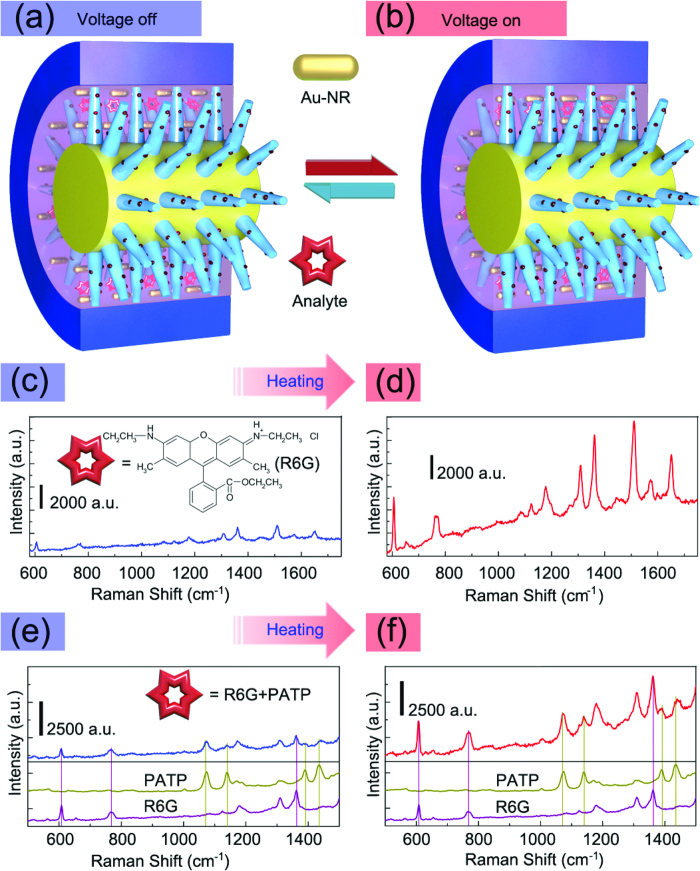
Thermo-responsive SERS detection of single-analyte and dual-analyte. (**a**,**b**) Schematic of the battery-controlled composite SERS system before and after voltage-on. (**c**,**d**) SERS spectra of 10^−7^ M R6G in response to voltage-off and voltage-on with an acquisition time of 30 s; (**e**,**f**) SERS spectra of PATP/R6G mixture (1.25 × 10^−5^ M: 1.25 × 10^−6^ M) in response to voltage-off and voltage-on. The lower SERS spectra in (**e**,**f**) are 5 × 10^−5^ M PATP and 5 × 10^−6^ M R6G respectively (acquisition time: 5 s). This figure was drawn by one of the coauthors (Qitao Zhou).

In the present SERS system, the mixed thermo-responsive pNIPAM microgels and Au-NRs surrounding around the Ag-NPs@ZnO-NTs, work as a temperature-sensitive trapping layer for the analytes. The constantan wire serves as not only a support for the Ag-NPs@ZnO-NTs but also a controller for the temperature-driven phase transformation of pNIPAM when the circuit is switched on. As shown in Fig. 4a–d, the SERS intensities increased significantly after the voltage was applied (Fig. 4c,d), which increased the enhancement factor almost 5 times, enabling the detection of a much lower concentration (10^–9^ M) of R6G (Fig. S4). This great enhancement was ascribed to the local field (|E|^2^) intensity enhancement in the smaller gaps between the neighboring Au-NRs and those between the Au-NRs and the nearby Ag-NPs on ZnO-NT^26^,^27^. The thermo-induced enhancement of local field intensity has been attested by surface enhanced fluorescence (SEF) of R6G. As shown in Movie S1 and Fig. S10 the fluorescence intensity increased so significantly that can be clearly identified by naked eyes after the voltage was applied. As the number of R6G molecules almost has not changed after the voltage was applied, the enhancement of fluorescence can be attributed to SEF based on the enhanced of local field intensity^28^,^29^. After replacing Au-NRs with Au-NPs at the same concentration, the corresponding temperature-sensitive SERS effect was not so obvious (Fig. S11). That was due to the fact that shrinking of pNIPAM microgels had a stronger effect on one-dimensional Au-NRs than zero-dimensional Au-NPs. The temperature change in the system was measured with an infrared thermometer (model 59 mini, Fluke, Norwich, UK) roughly (Fig. S12). Although the temperature on the surfaces of capillaries rather than in the capillaries was measured, the measured data were valuable as the temperature tends to be stable after voltage is switched on for several seconds.

The SERS intensity response to temperature was studied in the absence of pNIPAM (Fig. S13). It can be seen that the SERS intensity was reduced slightly after the voltage was applied, which was in agreement with the previous results^30^. This also confirmed that the SERS-intensity improvement was not caused by the temperature increase. Taking the collective effect, the battery-controlled composite SERS system can realize the well-controlled multi-type plasmonic couplings and effective trapping of the analytes, leading to high sensitivity of SERS detection.

In addition, this integrated SERS system has capability of simultaneous detection of multiple-analytes. Two analytes with different binding affinities were mixed with the pNIPAM and Au-NRs mixture, and then injected into the integrated SERS system. Figure 4e,f present the detection of both para-aminothiophenol (PATP) and R6G, where PATP can be adsorbed strongly on the Au-NR surface due to strong Au–S interaction while R6G has a weak interaction with the Au-NR surface. The SERS bands of both the analytes were observed in the spectra obtained before and after applying the voltage. For example, the 612, 774, 1179, 1362, 1310 cm^–1^ bands of R6G (purple), which were assigned to C–C–C ring in-plane, C–H out-of-plane bending, C–C stretching vibration, aromatic C–C stretching vibration and C=C stretching vibration of the R6G molecule, respectively^31^,^32^. These bands were readily distinguished from the 1077, 1142 and 1432 cm^–1^ shifts of PATP (dark yellow) assigned to a_1_ vibration mode and b_2_ vibration modes^33^. After the voltage was switched on, the intensity of all the bands increased almost 3 ~ 4 times. This indicated that the SERS intensity was modulated by strong temperature-driven trapping effect instead of the affinity of analyte with the SERS substrate. After switching on and off the battery for 4 cycles, the signal fluctuations were repeatable (Fig. S14), showing good stability of the SERS device. Except for the signal enhancement, the detection of the mixture solution with much lower concentration also has been realized (Fig. S15).

**Figure 5:**
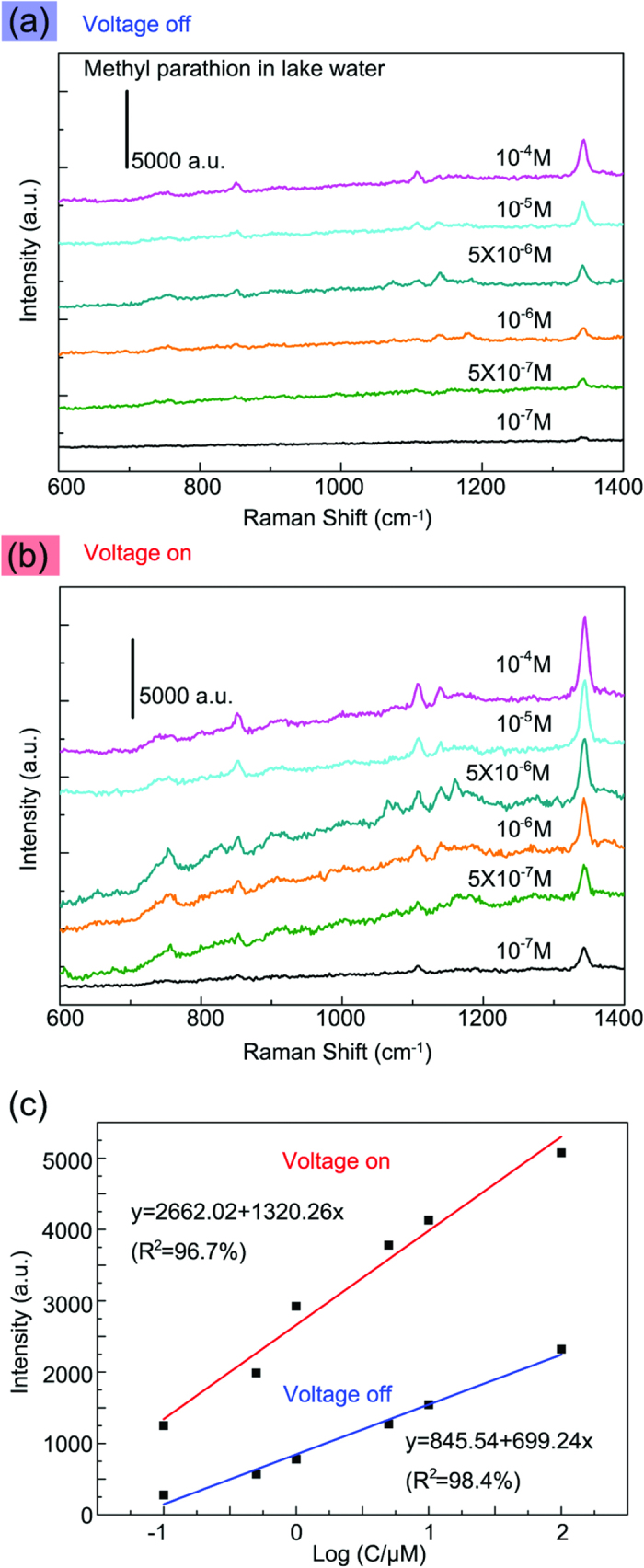
Thermo-responsive SERS detection of methyl parathion in lake water. (**a**,**b**) SERS spectra of different concentrations of methyl parathion in lake water before and after voltage-on. (**c**) The calibration curve of the integrated SERS systems before and after voltage-on.

To demonstrate the applicability of this integrated SERS system to real-world environmental samples, the integrated SERS system was employed to detect methyl parathion in lake water, as shown in Fig. 5 and Fig. S16. The pronounced bands at 851, 1108 and 1344 cm^–1^ were assigned to P–O stretching, C–N stretching and C–H bending in the methyl parathion molecule, respectively^34^, which provide the fingerprint proof for the analyte. Comparing Fig. 5a with Fig. 5b, it is found that switching on the voltage increased the intensity of all the SERS bands almost 3 ~ 4 times. Furthermore, the intensity of the fingerprint peak (1346 cm^–1^) exhibited a linear dependence on the logarithmic concentration of methyl parathion (Fig. 5c). The calibration curves were fitted as y = 845.54 + 699.24x (R^2^ = 98.4%) for the voltage-off case, and y = 2662.02 + 1320.26x (R^2^ = 96.7%) for the voltage-on case. Here, y is the SERS intensity at 1344 cm^–1^, and x is the logarithmic concentration of methyl parathion. The limit of detection (LOD) was estimated to be 10^–7^ M. This LOD was lower than the values of microfluidic-sensors reported previously^34^,^35^. The performance of the device can be further improved by replacing the glass capillary with quartz capillary that has better transparency. It should be noted that although the maximum enhancement was present for a 532 nm Raman laser for the FDTD simulations and PATP, a 633 nm laser was used for the Raman testing to avoid the photodegradation and the background fluorescence of R6G (Fig. S17 and Table S1).

In summary, the compact battery-controlled microfluidic SERS system exhibited high SERS activity due to multi-type plasmonic couplings, and had strong capability of trapping the analytes via the electric heating-driven thermo-responsive microgels. The high-density “hot spots” created in the compact 3D space was responsible for the high SERS enhancement. It is worth noting that the thermo-responsive microgels played an important role in amplifying the SERS signal. When pNIPAM microgels were collapsed by heating, the gaps among the plasmonic nanostructures were reduced, leading to stronger plasmonic field coupling between the nanostructures. This work has shown that the integrated SERS system has a great potential in real-time detection of aquatic pollutants.

## Methods Section

### Synthesis

Growth of cone-shaped ZnO-NT arrays on constantan wire: The constantan wire was washed sequentially with acetone, ethanol and DI water. The ZnO-NT arrays were then electrodeposited onto the constantan wire with a graphite sheet as the anode at a constant current density of 1 mA for 1 h in Zn(NH_3_)_4_(NO_3_)_2_ aqueous solution (100 mL, 0.05 M), which was prepared by gradually dropping ammonia water into Zn(NO_3_)_2_•6H_2_O aqueous solution until the solution became clear. The electrochemical cell was put into a water bath at 90 °C during the whole electrodeposition. Finally, the ZnO-NTs@constantan-wire was cleaned with DI water and dried for use.

Assembly of Ag-NPs on the ZnO-NTs@contantant-wire: The ZnO-NTs@contantant-wire was put in a 1 M aqueous AgNO_3_ solution for 30 min. Subsequent UV irradiation resulted in the formation of Ag-NPs on the surface of each ZnO-NT^36^. Then the Ag-NPs@ZnO-NTs@constantan-wire was inserted into the capillary.

Preparation of Au-NRs and Au-NPs: Au-NRs were synthesized by the seed-mediated growth method^37^. Au-NPs were synthesized with a trisodium citrate reduction method^38^. The as-prepared Au-NRs and Au-NPs were dispersed in desired amount of DI water after wash treatment.

Preparation of pNIPAM and Au-NRs mixture: 0.2 g of pNIPAM was dissolved into 10 mL of Au-NRs solution under magnetic stirring for 24 hrs.

Preparation of analyte samples: 5 × 10^−7^ M R6G was used to identify the SERS contribution from different building blocks. 2 × 10^−6^ M R6G was used as the SERS reporter in depth SERS mapping. For the thermo-responsive SERS samples, 200 μL aliquots of Au-NR and pNIPAM mixture were stabilized at 4 °C, and 200 μL of analyte was added to each of the Au-NR and pNIPAM mixture to achieve 10^−7^ M R6G, PATP/R6G (1.25 × 10^−5^ M: 1.25 × 10^−6^ M) respectively, and then injected into the capillary. Methyl parathion contaminated lake water was obtained by dissolving different amount of methyl parathion in lake water taken from Dongpu Lake.

### Characterization

The morphology of the resultant products was characterized by a field-emission SEM (Hitachi S-4800) and energy dispersive X-ray spectroscopy (EDS, Oxford). TEM images of the individual products were taken under a JEM-2010 (JEOL, Japan).

### SERS Measurements

The excitation wavelength was 633 nm from a HeNe laser. The SERS spectra were recorded on a confocal microprobe Raman system (Renishaw, inVia). The voltage applied to the constantan wire was 4.8 V.

### FDTD simulations

FDTD simulations were performed to study the electromagnetic field distribution using the commercially available Optiwave software. The experimental configuration was replicated using the characterization data when possible. For the microgel after heating several possibilities were tested, with the largest enhancement assumed to give the largest increase to the signal. A simulation cell with a grid size of 0.5 nm was used. The length and diameter of the Au-NR were 50 nm and 10 nm respectively. As shown in Fig. 2b, the Ag-NPs have two main sizes. For each kind we picked 10 nanoparticles randomly and figured out their average diameters. The bigger one has an average diameter around 20 nm and the smaller one has an average diameter around 10 nm. In the following simulations both situations have been considered. The experimental UV-Vis absorption was first matched using a full-spectrum simulation, then a monochromatic field at 633 nm was used to see the EM field enhancement from the laser. The refractive index for gold and silver was matched to the data of Palik^39^. ZnO-NTs were given a refractive index of 2.03. A plane-wave source with both X and Y polarization was used. The refractive index of the microgel changes with heating^22^.

### Thermo-induced SEF

To obtain the analyte sample, 200 μL of R6G solution (10^−3^ M) was added to 200 μL Au-NR and pNIPAM mixture. The compact battery-controlled microfluidic SERS system was placed under a UV lamp (Fig. S10a). Then the analyte solution was injected into the system and the UV lamp was switched on. The fluorescence changing processes before and after heating have been recorded by normal camera (Fig. S10b,c and Movie S1). In order to identify the change of fluorescence intensities more clearly, the fluorescence images were obtained using a high-resolution wide field fluorescent microscope (Leica DMI 3000B). As shown in Fig. S10d to S10i the changes of fluorescence were distinct and repeatable. The temperature quenching of fluorescent molecules such as R6G has been widely accepted^40^. That is to say, the fluorescence improvement was not caused by the temperature increase directly. Our results were also counter to the further work, which using Au-NPs@pNIPAM core@shell colloids as temperature response fluorescence system. When the Au-NPs@pNIPAM core@shell colloids system being heated, the shrinking of pNIPAM molecule will drive the analyte molecule very close to the Au-NPs core, the fluorescence will be quenched^18^. In the compact battery-controlled microfluidic SERS system, when the voltage is switched on the shrinking of pNIPAM molecule will drive part of the R6G molecules much closer to the plasmonic Au-NRs, leading to the fluorescence quenching of these R6G molecules. On the other hand, the shrinking of pNIPAM molecule also abridge the spacing of the neighboring Au-NRs in the microgels, and that of the Au-NRs and their nearby Ag-NPs decorated on the ZnO-NTs, leading to the formation of high-density “hot spots” in the three-dimensional (3D) space with much improved local field intensity. Thus, the fluorescence intensities from the rest of R6G molecules have been enhanced so greatly that can offset the quenching of high temperature and Au-NRs.

## Additional Information

**How to cite this article**: Zhou, Q. *et al.* A Surface-Enhanced Raman Scattering Sensor Integrated with Battery-Controlled Fluidic Device for Capture and Detection of Trace Small Molecules. *Sci. Rep.* **5**, 12865; doi: 10.1038/srep12865 (2015).

## Supplementary Material

Supplementary Information

Supplementary Movie S1
