# Supplementary material for: A Surface-Enhanced Raman Scattering Sensor Integrated with Battery-Controlled Fluidic Device for Capture and Detection of Trace Small Molecules
Source: Sci Rep. 2015 Aug 4;5:12865. doi: 10.1038/srep12865 (PMC4523941; doi:10.1038/srep12865)
Supplement: Supplementary Information [file srep12865-s1.pdf]

Supplementary Information

A Surface-Enhanced Raman Scattering Sensor Integrated with Battery-Controlled Fluidic Device for Capture and Detection of Trace Small Molecules

Qitao Zhou,[1] *Guowen Meng,*[1,2,*] *Peng Zheng,*[3] *Scott Cushing,*[4] *Nianqiang Wu,*[3] *Qing Huang,*[5] *Chuhong Zhu,*[1] *Zhuo Zhang,*[1] *and Zhiwei Wang*[1]

[1]Key Laboratory of Materials Physics, and Anhui Key Laboratory of Nanomaterials and Nanostructures, Institute of Solid State Physics, Chinese Academy of Sciences, Hefei 230031, P. R. China

[2]University of Science and Technology of China, Hefei, 230026, China

[3]Department of Mechanical & Aerospace Engineering, West Virginia University, P.O. Box 6106, Morgantown, WV 26506, USA

[4]Department of Physics and Astronomy, West Virginia University, Morgantown, WV 26506, USA

[5]Key Laboratory of Ion Beam Bioengineering, Chinese Academy of Sciences, Hefei 230031 (P. R. China)

*To whom correspondence should be addressed.

Tel: (86) 0551-65592749, Fax: (86) 0551-65591434, E-mail: gwmeng@issp.ac.cn.

The Supporting Information includes:

Figures and table
References

Figures and table

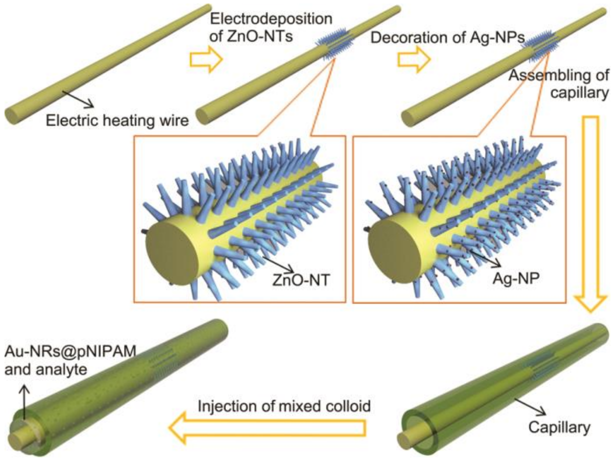

Figure S1. Schematic illustration of fabrication of the battery-controlled composite SERS system. This figure was drawn by one of the coauthors (Qitao Zhou).

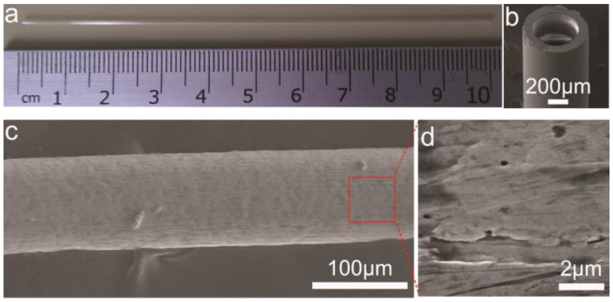

Figure S2. Characterization of capillary and constantan wire. (a) Optical photo of the capillary. (b) SEM image of the cross-section of capillary. (c) and (d) SEM images of the constantan wire and the close-up view.

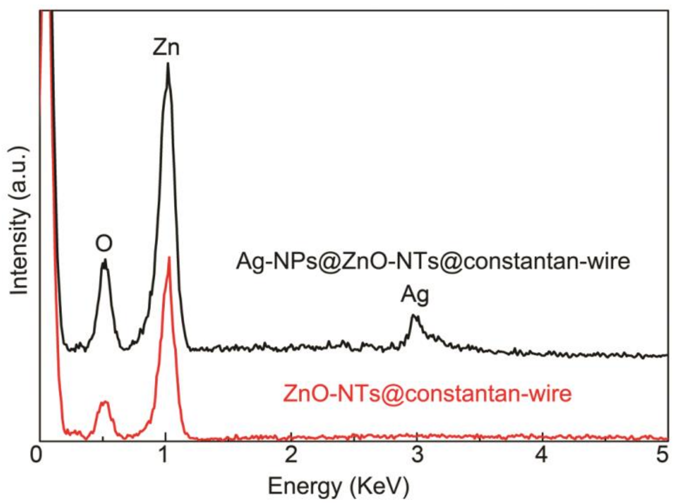

Figure S3. EDS spectra of the ZnO-NTs before and after decorated with Ag-NPs.

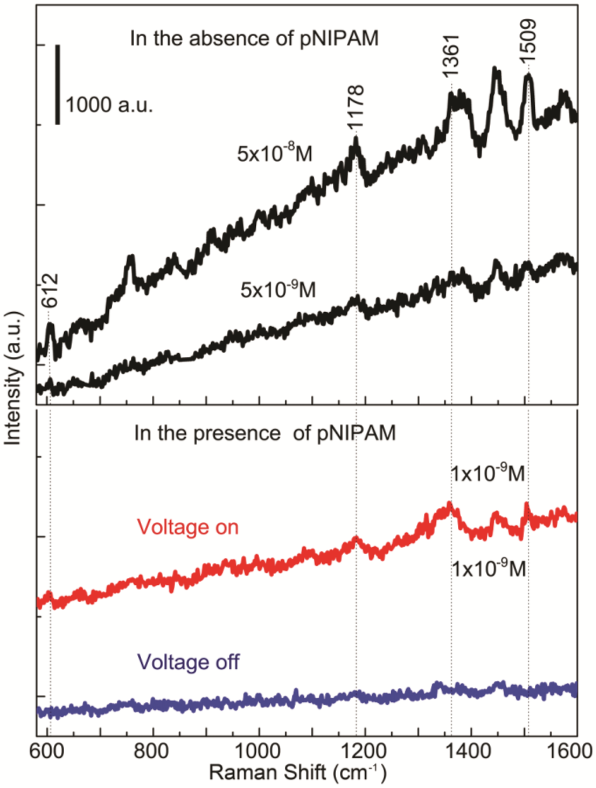

Figure S4. The SERS activities in the absence of pNIPAM and in the presence of pNIPAM. The SERS spectra of different concentrations R6G solutions (5×10^{-8} M and 5×10^{-9} M) obtained by the system in the absence of pNIPAM; lower concentration (10^{-9} M) of R6G can be detected after electrifying the constantan wire in the presence of pNIPAM.

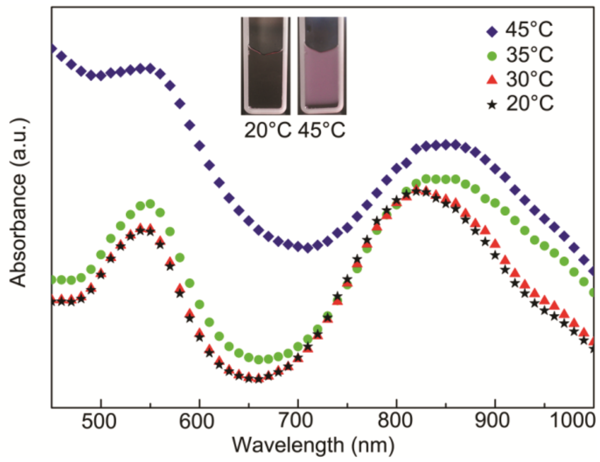

Figure S5. UV-Visible spectra for Au-NRs and pNIPAM mixture at different temperatures (20, 30, 35, and 45 ^{o}C).

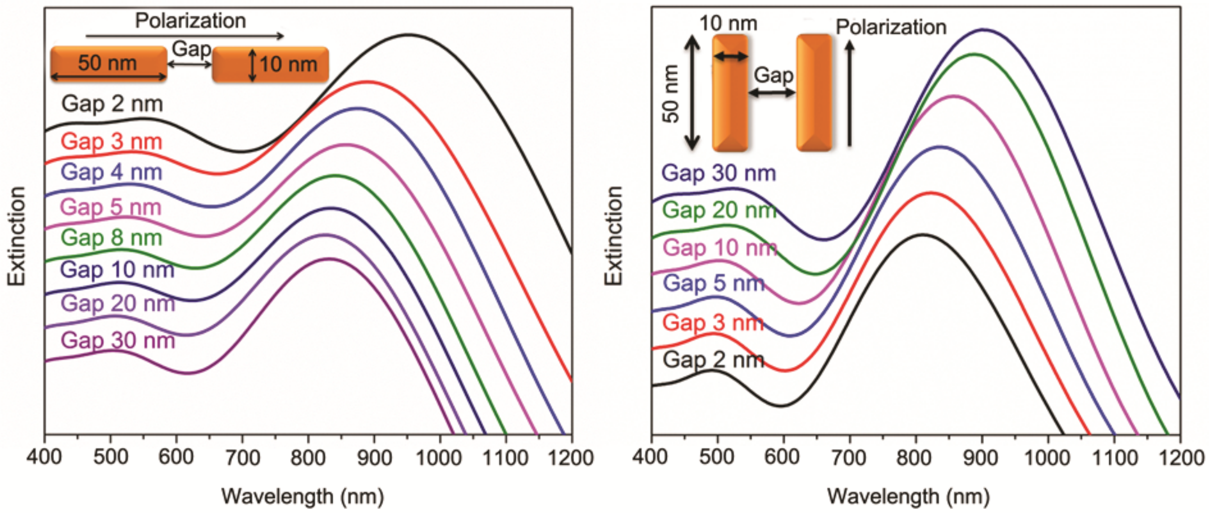

Figure S6. Calculated UV-Vis extinction spectra for Au-NRs in end-to-end and parallel configurations with various separation distances. The end-to-end configuration shows a red-shift of the LSPR peak centered at ~850 nm, consistent with experiment. The arrow shows the polarization of the input field.

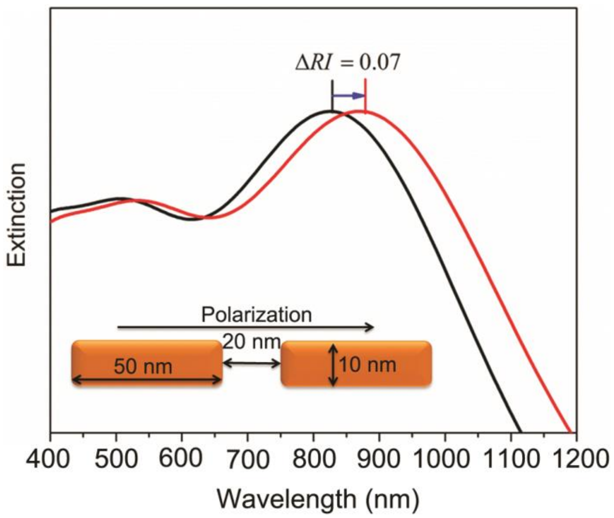

Figure S7. Calculated UV-Vis extinction spectra with heating of the microgel but no shrinking. When heated, the microgel refractive index shifts 0.07[1], inducing a 50 nm red shift of the extinction peak for the Au-NRs. The arrow shows the polarization of the input field.

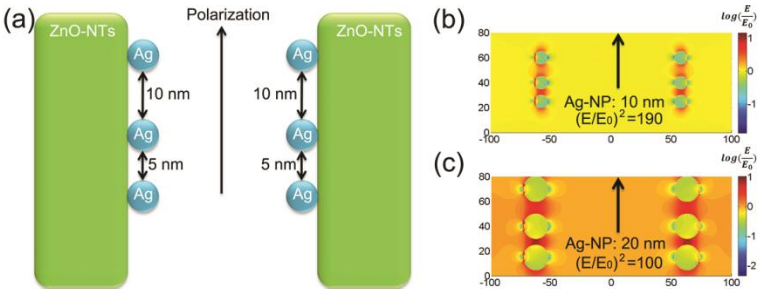

Figure S8. FDTD simulations of the electric field distributions for ZnO-NTs decorated with Ag-NPs. (a) The arrow shows the polarization of the input field. Electromagnetic field enhancement for (b) 10 nm and (c) 20 nm Ag-NP is shown on a log-scale.

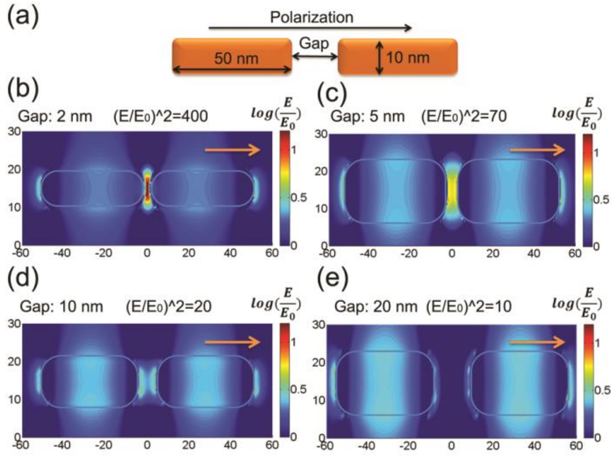

Figure S9. FDTD simulations of the electric field distributions for Au-NRs with various gap distances. (a) The direction of the input polarization to the NP axis. The $|E/E_0|^2$, the field enhancement normalized to the input field, for a (b) 2 nm gap, (c) 5 nm gap, (d) 10 nm gap, and (e) 20 nm gap is shown on a log-scale. The gold arrow shows the polarization of the input field.

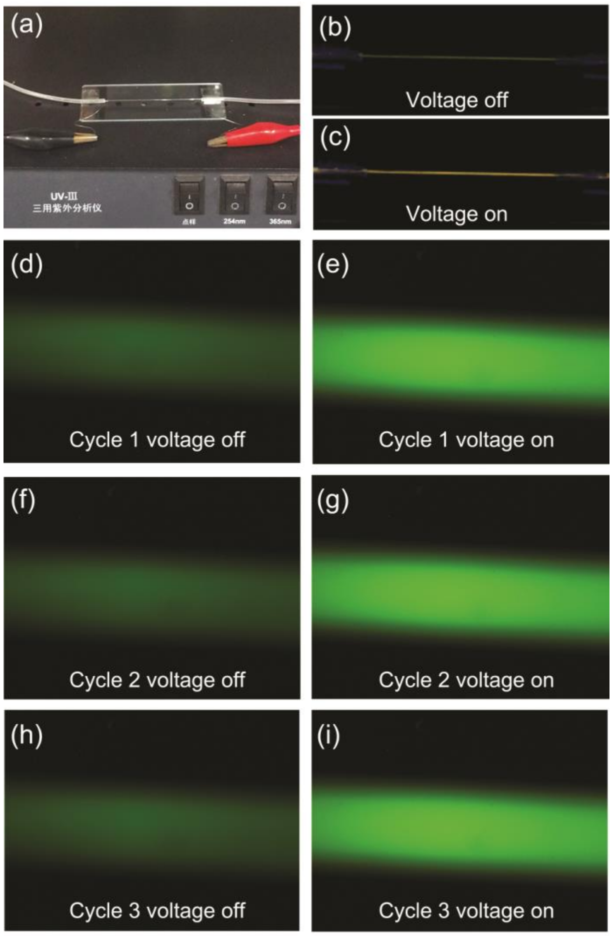

Figure S10. The thermo-induced surface enhanced fluorescence (SEF). (a) Optical photo of the SERS system placed under a UV lamp. (b) and (c) the optical photos of the system with the voltage off and on after injection of analyte solution and under UV irradiation. (d) to (i) The fluorescence images of different heating cycles.

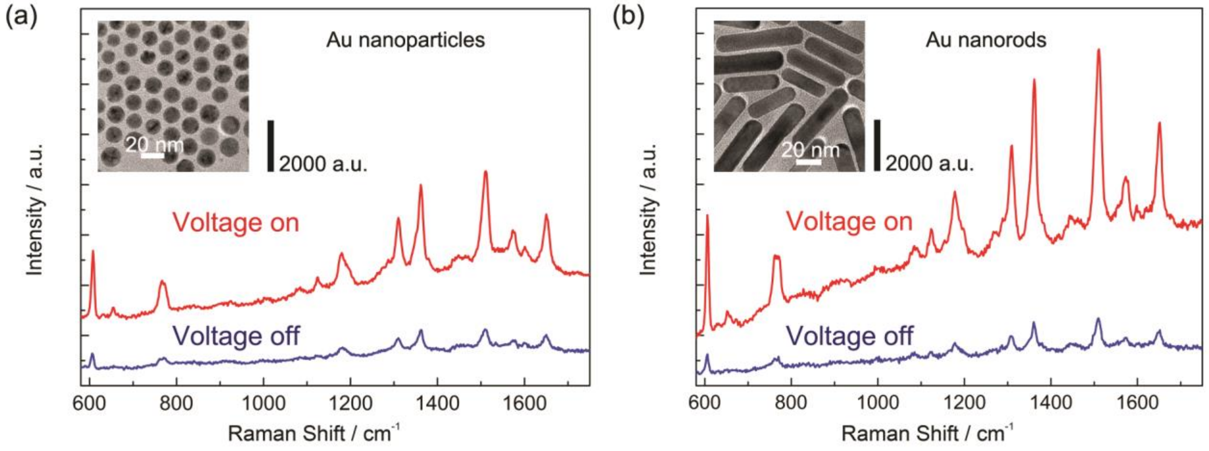

Figure S11. (a) SERS spectra of 10^{-7} M R6G in response to voltage-off and voltage-on with Au-NPs as plasmonic structures in the mixture. (b) SERS spectra of 10^{-7} M R6G in response to voltage-off and voltage-on with Au-NRs as plasmonic structures in the mixture. The concentrations of Au-NPs and Au-NRs used in the experiment were almost the same. All spectra were acquired with an integration time of 30 s.

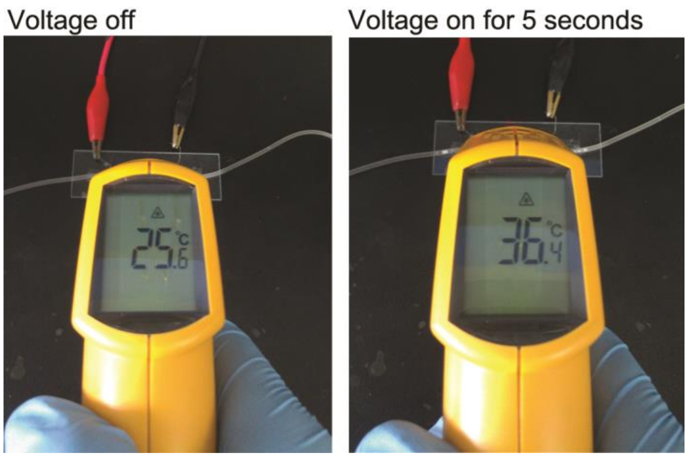

Figure S12. The temperature change measured with an infrared thermometer. Before voltage on, the temperature was 25.6 °C. After voltage on for 5 s, the temperature was 36.4 °C and tended to stable.

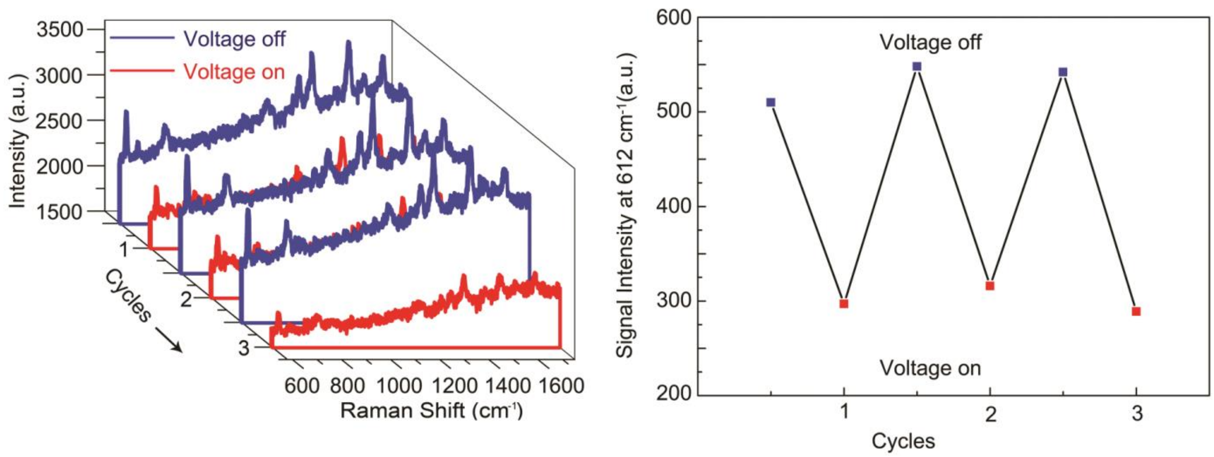

Figure S13. The response of the SERS intensities to temperature under the alternation of voltage in the absence of pNIPAM. The concentration of R6G solution is 10^{-7} M. All spectra were acquired with an integration time of 5 s.

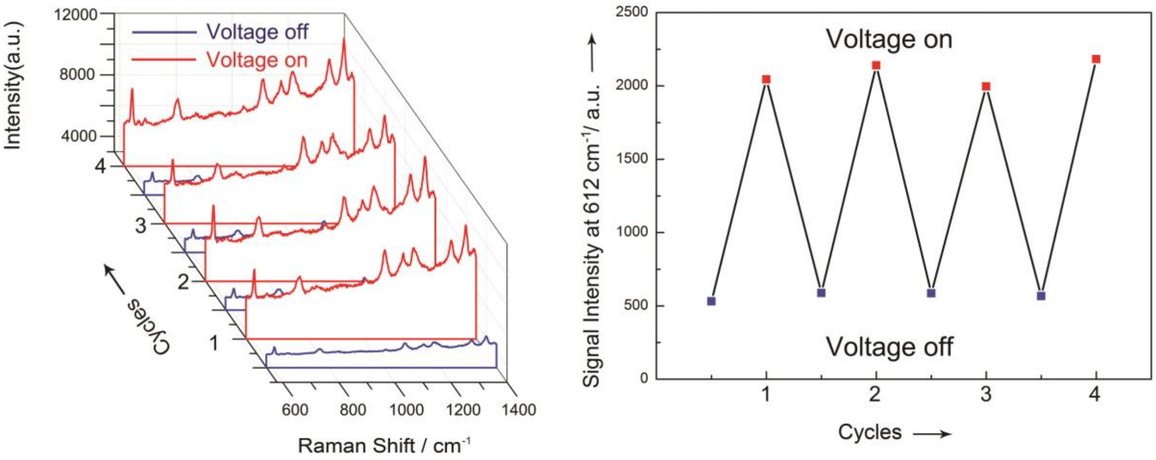

Figure S14. The response of the SERS intensities to temperature change by modulation of voltage in the presence of pNIPAM. The PATP/R6G mixture (1.25×10^{-5} M: 1.25×10^{-6} M) has been used in the detection. All spectra were acquired with an integration time of 5 s.

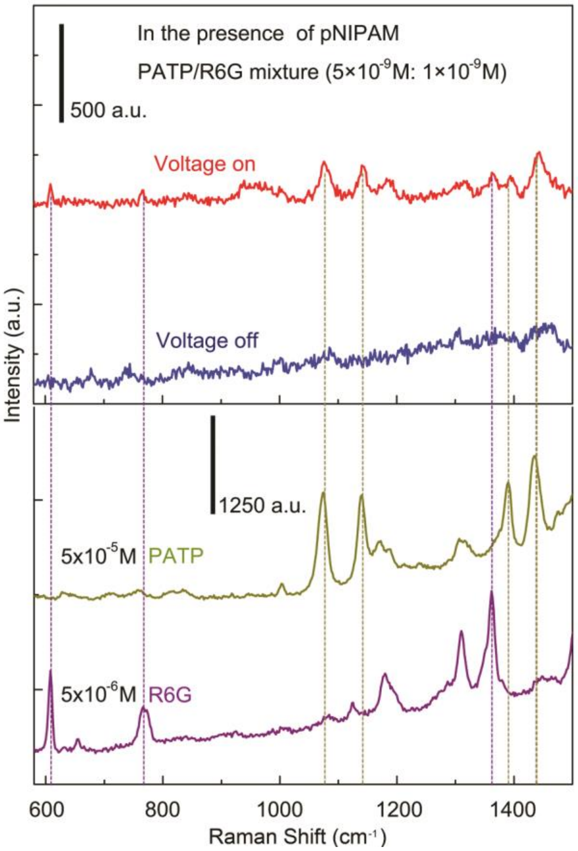

Figure S15. A Lower concentration of PATP/R6G mixture (5×10^{-9} M: 1×10^{-9} M) can be detected after electrifying the constantan wire in the presence of pNIPAM. The lower SERS spectra were 5×10^{-5} M PATP and 5×10^{-6} M R6G, respectively (acquisition time: 5 s).

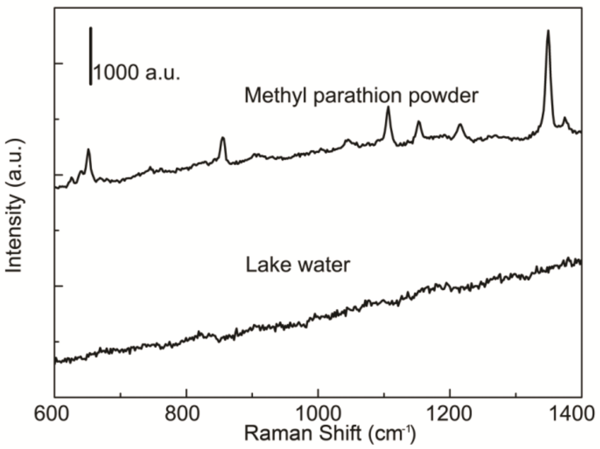

Figure S16. The SERS spectrum of lake water, and the Raman spectrum of solid methyl parathion.

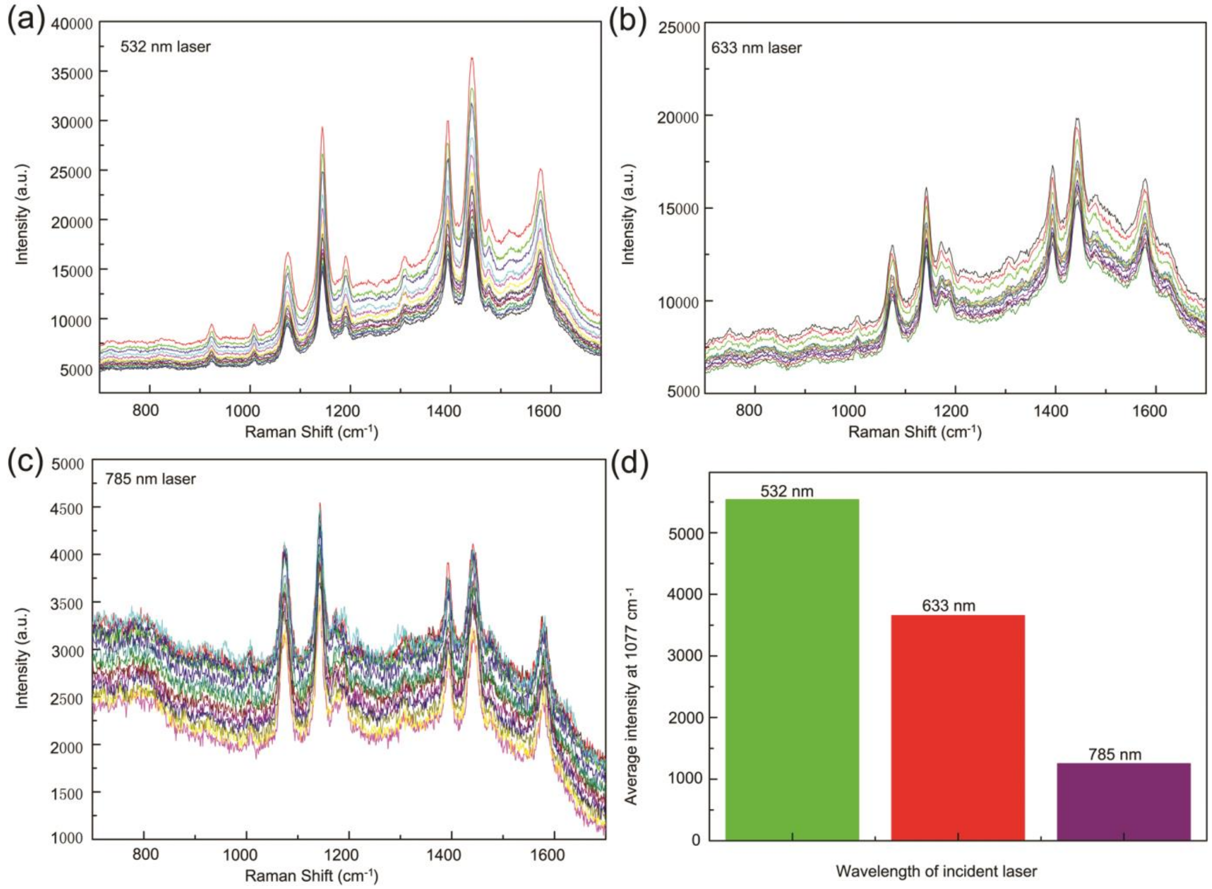

Figure S17. 10^{-4} M PATP was used to identify the influence of different wavelengths of incident lasers. The influence of different wavelengths of incident lasers has been studied, as shown in Fig. S17. 14 random spots across the system were examined under the three incident lasers of (a) 532 nm (10 mW, integration time: 5 s), (b) 633 nm (8.5 mW, integration time: 5 s) and (c) 785 nm (12.5 mW, integration time: 5 s) wavelengths. (d) The average band intensity at 1077 cm^{-1} under excitation by different wavelengths of incident lasers.

Table S1.

The simulated enhancement factor when excited by different lasers and the corresponding experiment as shown in Fig. S17.

**Laser**	**Max** $(\frac{E_{ext}}{E_0})^2$	**Max** $(\frac{E_{emi}}{E_0})^2$	**Max Raman Enhancement factor** $[(\frac{E_{ext}}{E_0})^2(\frac{E_{emi}}{E_0})^2]$	**Average intensity at 1077 cm^{-1}**
532 nm	2000	1580	3.16e+06	5536
633 nm	1700	1180	2.01e+06	3655
785 nm	500	280	1.40e+05	1252

References

1. Garner, B. W., Cai, T., Ghosh, S., Hu, Z., Neogi, A. Refractive index change due to volume-phase transition in polyacrylamide gel nanospheres for optoelectronics and bio-photonics. *Appl. Phys. Express.* **2**, 057001 (2009).
